# Sustained low disease activity measured by ASDAS slow radiographic spinal progression in axial spondyloarthritis patients treated with TNF-inhibitors: data from REGISPONSERBIO

**DOI:** 10.1186/s13075-021-02695-5

**Published:** 2022-01-21

**Authors:** Maria Llop, Mireia Moreno, Victoria Navarro-Compán, Xavier Juanola, Eugenio de Miguel, Raquel Almodóvar, Eduardo Cuende Quintana, Jesús Sanz Sanz, Emma Beltrán, M. Dolores Ruiz Montesinos, Joan Calvet, Antoni Berenguer-Llergo, Jordi Gratacós, the Regisponserbio group, Pedro Zarco Montejo, Beatriz Joven, Miriam Almirall, Ma Cruz Fernandez Espartero, Enrique Batlle Gualda, Cristina Campos, Eduardo Collantes Estevez, Pilar Font, Teresa Clavaguera Poch, Luis F. Linares Ferrando, Carlos Rodríguez Lozano, Beatriz Yoldi

**Affiliations:** 1 Rheumatology, Parc Taulí Hospital Universitari, I3PT Research Institute (UAB), Universitat Autónoma de Barcelona (UAB), Sabadell, 08208 Spain; 2 Rheumatology, Hospital Universitario La Paz, Madrid, Spain; 3 Rheumatology, Hospital Universitari Bellvitge, L’Hospitalet de Llobregat, Barcelona, Spain; 4 Rheumatology Unit, Hospital Alcorcon Foundation, Madrid, Spain; 5 Rheumatology, Hospital Universitario Príncipe de Asturias, Alcalá de Henares, Spain; 6 Rheumatology, Hospital Universitario Puerta de Hierro, Madrid, Spain; 7 Rheumatology, Hospital del Mar, Barcelona, Spain; 8 Rheumatology, Hospital Universitario Virgen Macarena, Sevilla, Spain; 9 I3PT Research Institute (UAB), Sabadell, Spain

**Keywords:** Spondyloarthritis, Radiology, Biological therapies, Inflammation, Outcome measures

## Abstract

**Background:**

To evaluate the influence of the disease activity on radiographic progression in axial spondyloarthritis (axSpA) patients treated with TNF inhibitors (TNFi).

**Methods:**

The study included 101 axSpA patients from the Spanish Register of Biological Therapy in Spondyloarthritides (REGISPONSERBIO), which had clinical data and radiographic assessment available. Patients were classified into 2 groups based on the duration of TNFi treatment at baseline: (i) long-term treatment (≥4 years) and (ii) no long-term treatment (< 4 years). Radiographs were scored by two readers according to the modified Stoke Ankylosing Spondylitis Spine Score (mSASSS) with known chronology. Disease activity differences between patients’ groups at each time point were assessed using a linear mixed-effect model.

**Results:**

Radiographic progression was defined as an increase in ≥2 mSASSS units**.** At inclusion, approximately half of the patients (45.5%) were receiving long-term treatment with TNFi (≥4 years). In this group of subjects, a significant difference in averaged Ankylosing Spondylitis disease Activity Score (ASDAS) across follow-up was found between progressors and non-progressors (2.33 vs 1.76, *p*=0.027, respectively). In patients not under long-term TNFi treatment (54.5%) though, no significant ASDAS differences were observed between progressors and non-progressors until the third year of follow-up. Furthermore, no significant differences were found in progression status, when disease activity was measured by Bath Ankylosing spondylitis Disease Activity Index (BASDAI) and C reactive protein (CRP).

**Conclusions:**

Patients on long-term TNFi treatment with a mean sustained low disease activity measures by ASDAS presented lower radiographic progression than those with active disease.

**Supplementary Information:**

The online version contains supplementary material available at 10.1186/s13075-021-02695-5.

## Introduction

Axial spondyloarthritis (axSpA) is a chronic inflammatory disease with predominantly axial symptoms, such as inflammation of the sacroiliac joints and spine, leading to structural damage [1]. AxSpA can be classified as into radiographic SpA (r-axSpA, also known as ankylosing spondylitis (AS)) or non-radiographic SpA (nr-axSpA) depending on the presence or absence of definite radiographic sacroiliitis respectively, according to the modified New York criteria grading system [2].

Inflammation, when persists, leads to structural damage, which can be detected on conventional radiograph as sclerosis, erosions, and new bone formation (syndesmophytes). A major concern in the SpA field is the development of new bone formation in the spine due to its contribution to disease severity [3]. While the clinical efficacy of TNF inhibitors (TNFi) in axSpA has been widely shown to decrease symptoms and signs of the disease in randomized clinical trials, it is still unclear whether TNFi inhibits radiographic progression. According to the early data published from the pivotal studies, TNFi did not seem to inhibit radiographic damage in axSpA [4–6]. Nevertheless, some subsequent studies suggested that long-term treatment with TNFi (more than 4 years) could slow down radiographic progression in axSpA [7, 8]. However, more data is needed to draw further conclusions.

More recently, the Swiss Clinical Quality Management cohort [9] reported their results in patients with r-axSpA treated with TNFi who underwent radiographic assessments every 2 years during a 10-year period. The study showed that radiographic spinal progression was nearly entirely inhibited in the following 2-year radiographic interval in patients with r-axSpA who achieved an inactive disease status (AS Disease Activity Score (ASDAS) ≤1.3) when receiving TNFi. Currently, the ASAS-EULAR management recommendations for axSpA are based on a treat-to-target approach [10]. However, in clinical practice, it might be quite challenging to achieve inactive disease in axSpA patients and low disease activity seem to be a more realistic target [11].

This study aimed to evaluate the influence of low disease activity measured by ASDAS (< 2.1) on radiographic progression in axSpA patients treated with TNFi, using the data from REGISPONSERBIO.

## Methods

### Patients and study design

**Fig. 1:**
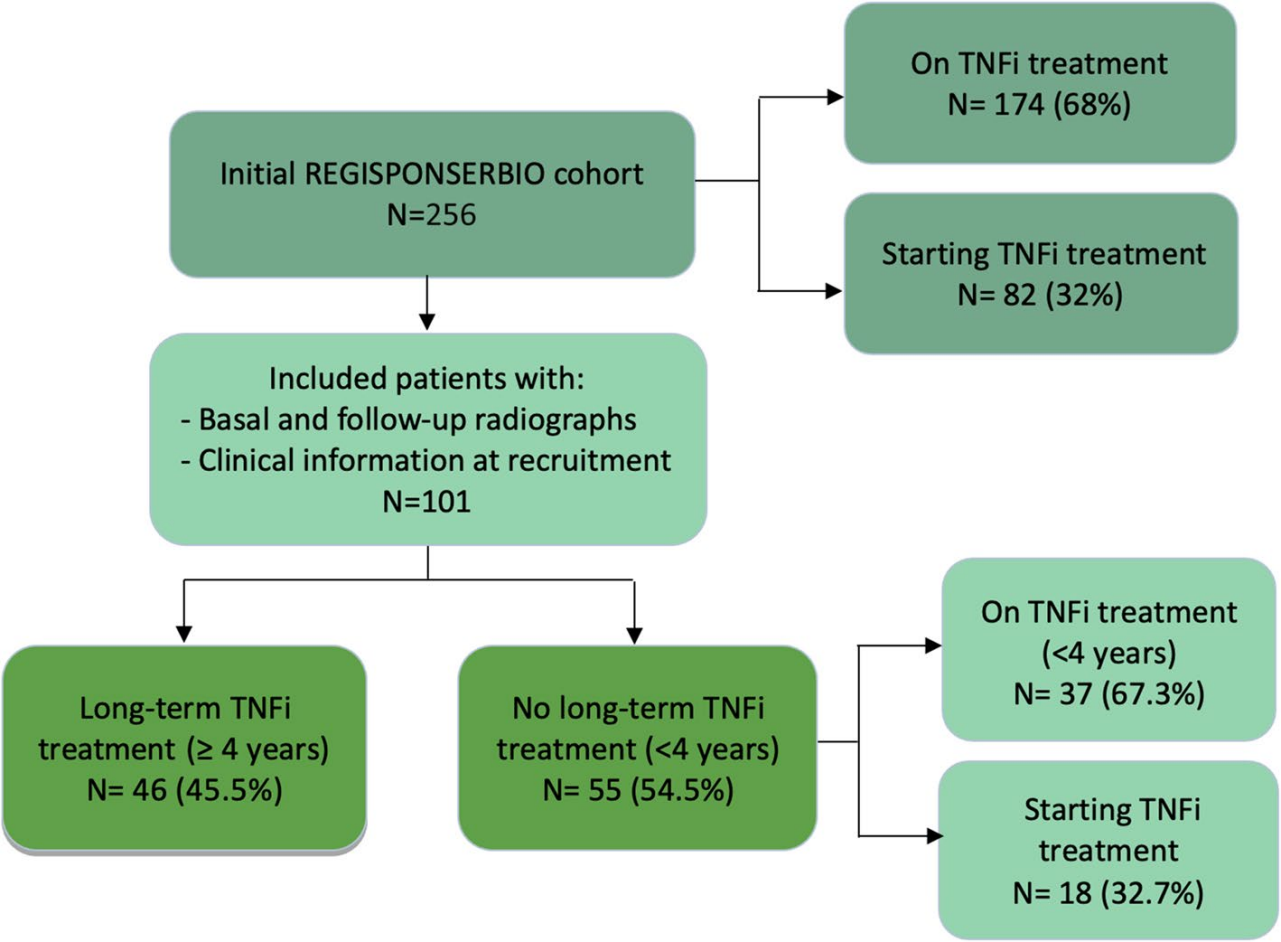
Flow chart describing the inclusion of REGISPONSERBIO patients in our study

The study population comprised all patients included in REGISPONSERBIO (Spanish Registry of axSpA under TNFi therapy) who presented at least 2 sets of cervical and lumbar spinal radiographs, one at inclusion and one after a minimum interval of 2 years. A detailed description of the entire cohort has been reported previously [12]. Briefly, REGISPONSERBIO is a prospective multicenter cohort design study that includes both patients who were already receiving TNFi treatment and those who started on TNFi therapy at the time of recruitment (Fig. 1). Patients in the registry were followed up for a 3-year period in which they underwent clinical and laboratory controls every 6 months. Criteria for starting TNFi therapy was made according to the recommendations of the Spanish Society of Rheumatology [13]. The register and subsidiary efficacy and safety analyses complied with the Declaration of Helsinki and were approved by the Ethical Review Boards of all participating hospitals, and patients signed an informed consent form to be included.

For this study, patients were classified into 2 groups based on the duration of TNFi treatment at recruitment: (i) long-term duration (patients receiving TNFi for at least 4 years) and (ii) no long-term duration (patients receiving TNFi for less than 4 years and patients who started TNFi at study entry). This cut-off was selected according to previously published evidence for the potential influence of TNFi on radiographic progression in axSpA from various studies [7–9].

### Assessment of radiographic progression

Spinal radiographs were collected centrally in the rheumatology department of Hospital Parc Taulí. The images were anonymized and scored in random order by 2 independent and trained readers (ML and MM), who were blinded to all clinical data but aware of the chronological order. The images were scored according to the modified Stoke Ankylosing Spondylitis Spinal Score (mSASSS) [14]. According to the mSASSS, anterior corners of the vertebral bodies from lower C2 to upper T1 (cervical spine) and from lower T12 to upper S1 (lumbar spine) were scored as follows: 0 = normal; 1 = erosion, sclerosis, and/or squaring; 2 = non-bridging syndesmophyte; and 3 = bridging syndesmophyte, giving a range for the entire score from 0 to 72. No adjudication was performed. The final mSASSS score included in the analysis was calculated as a mean of the mSASSS scores of both readers. Syndesmophytes were considered present if both readers assigned a score of ≥2 to a vertebral corner (VC). A change of the mSASSS score by ≥2 points was taken as the definition of progression [9, 15].

Only scores from radiographs with ≤ 3 missing VC per cervical or lumbar segment were used. Missing scores of single VCs were substituted with scores for the respective vertebral corners at other time point, if available, or with a score of zero if scores for both time points were missing [16, 17].

### Disease activity

Disease activity measurements included the Bath Ankylosing Spondylitis Disease Activity Index (BASDAI), ASDAS-CRP, and C reactive protein (CRP) serum levels. These were collected at start of TNFi, at start of the study (recruitment) and subsequently every 6 months over a follow-up period of 3 years. Disease activity states were assigned using the ASDAS cut-off levels [18]: inactive disease (ASDAS <1.3), low disease activity (1.3 ≤ASDAS <2.1), high disease activity (2.1 ≤ASDAS ≤3.5), and very high disease activity (ASDAS >3.5). Active disease was defined as ASDAS ≥2.1 or, alternatively, BASDAI ≥4.

### Statistical analysis

Concordance in radiographic assessment between both readers was explored by calculating the intraclass correlation coefficient (ICC, two-way mixed, single score method) and Cohen’s kappa coefficient for continuous and categorical variables, respectively. Additionally, a Bland-Altman plot was drawn, and the smallest detectable change (SDC) was determined to assess the sensitivity to change [19].

For description purposes, continuous variables were described by their median and minimum and maximum values, while absolute and relative frequencies were used for categorical parameters. Differences between included and excluded patients from the RESPONGISERBIO cohort were evaluated regarding variables potentially linked to radiographic progression, using the Mann-Whitney and Fisher tests for continuous and categorical variables, respectively. To assess differences between patient groups, ASDAS, BASDAI, and CRP were fitted to linear mixed-effects models that included a random intercept in order to model the patient’s effect, and a fixed effect coding the time point for which the measurement had been taken. Patient group was included in the models as an explanatory variable, which described the individuals’ characteristics according to their treatment status and their radiographic progression in four subsets: (i) patients not under long-term TNFi treatment without radiographic progression, (ii) patients not under long-term TNFi treatment with radiographic progression, (iii) patients under long-term TNFi treatment without radiographic progression, and (iv) patients under long-term TNFi treatment with radiographic progression. This model parametrization is mathematically equivalent to include the treatment status and the radiographic progression as covariates, together with the interaction of these two parameters. As potential confounding factors, we considered the length of the radiographic interval and the following patients’ characteristics collected at the time of recruitment: gender, age, smoking habit, radiographic sacroiliitis, mSASSS average, presence of syndesmophytes, HLAB27, and time from onset of symptoms. These variables were included in the models as fixed effects to obtain adjusted estimates of disease activity differences and group means. Binarized versions of these disease activity measurements were analyzed similarly, using generalized linear mixed-effects models with a binomial distribution (logistic regression). The association between mSASSS and ASDAS increases relative to baseline values was also assessed using a standard linear model (no random effects included). When necessary, a Tukey’s transformation ladder was applied to continuous variables in order to fulfill the assumptions of the linear model (lambda parameters: 0.5 for time from symptomatology onset; 0.25 for CRP; 0 for MSASSS increase at 3 years follow-up compared to baseline). For analyses comparing the patients groups of interest, mean differences, adjusted means (for continuous outcomes), odds ratio (OR, for binary outcomes), and their corresponding standard errors were used to measure the magnitude of effects. As long-term TNFi-treated patients kept essentially the same disease activity levels during the follow-up, differences across follow-up in these patients were assessed by averaging time point within progression groups. Adjusted means were computed using our own dataset as reference population. Statistical significance was assessed using Wald tests. All analyses were carried out using R [20].

## Results

A total of 256 patients were included in REGISPONSERBIO, of whom 101 with axSpA were included in the analysis based on the availability of full sets of radiographs (lumbar and cervical lateral view) and clinical data. Of these patients, 46 (45.5%) were classified in the long-term TNFi group (≥4 years) and 55 (54.5%) in the not long-term group (<4 years). Radiographic progression was observed in 32.6% of the long-term TNFi patients and a 27.3% for not long-term treatment (15 patients in each group). Demographic, clinical, and radiographic data of the patients at the time of the first radiograph are shown in Table 1 and Supplementary Tables S1 and S2 and were similar to those showed by the REGISPONSERBIO patients not included in the study (Supplementary Tables S3 and S4), mostly differing in the duration of TNFi treatments. This time point coincided with the onset of TNFi in 18 (18%) patients. At inclusion, 52.1% patients had low disease activity status (ASDAS <2.1), and only 24% of these had inactive disease (Table 1). Association with radiographic progression was explored specifically in each patient group defined by their treatment status at recruitment (TNFi ≥4 years or TNFi <4 years).

**Table 1:** Baseline characteristics and radiographic progression of the 101 patients included in the study by baseline TFNi treatment status.

	N		TNFi treatment ≥ 4years ***n***=46 (45.5%)	TNFi treatment < 4years ***n***=55 (54.5%)	All ***n***=101
Age, years	101		46.0 (25.0, 75.0)	47.0 (21.0, 75.0)	46.0 (21.0, 75.0)
Female, sex %	101		8 (17.4%)	11 (20.0%)	19 (18.8%)
Radiographic progression (mSASSS ≥ 2)	101		15 (32.6%)	15 (27.3%)	30 (29.7%)
HLA-B27 positive, %	99		43 (95.6%)	43 (79.6%)	86 (86.9%)
AS, %	101		41 (89.1%)	45 (81.8%)	86 (85.1%)
BMI	95		26.7 (19.8, 40.8)	25.4 (19.4, 32.0)	26.0 (19.4, 40.8)
Current smokers, %	101		16 (34.8%)	15 (27.3%)	31 (30.7%)
Symptom duration, years	97		19.0 (2.0, 52.0)	11.0 (0.0, 50.0)	15.0 (0.0, 52.0)
CRP (mg/L)	94		2.9 (0.10, 26.0)	4.3 (0.0, 88.7)	3.4 (0.0, 88.70)
CRP < 5	94		27 (62.8%)	26 (51.0%)	53 (56.4%)
BASDAI (0-10)	97		3.0 (0.2, 8.6)	4.2 (0.40, 8.8)	3.2 (0.20, 8.8)
BASDAI < 4	97		30 (66.7%)	24 (46.2%)	54 (55.7%)
ASDAS-CRP	96		1.71 (0.2, 5.1)	2.3 (0.3, 5.1)	2.1 (0.2, 5.1)
Low disease (ASDAS < 2.1), %	96		28 (63.6%)	22 (42.3%)	50 (52.1%)
Inactive disease (ASDAS <1.3), %	96		10 (22.7%)	13 (25.0%)	23 (24.0%)
BASFI (0–10)	98		3.6 (0.0, 9.1)	4.2 (0.0, 9.3)	3.9 (0.0, 9.3)
BASMI (0–10)	82		2.9 (0.5, 7.3)	3.0 (0.6, 6.4)	2.9 (0.5, 7.3)
mSASSS (0–72)	101		10.0 (0.0, 72.0)	3.0 (0.0, 66.0)	5.0 (0.0, 72.0)
Syndesmophytes present, %	101		26 (56.5%)	25 (45.5%)	51 (50.5%)
On NSAID treatment, %	99		23 (51.1%)	34 (63.0%)	57 (57.6%)
On TNFi treatment, %	100		45 (100.0%)	37 (67.3%)	82 (82.0%)
Number of previous TNFi	0	101	24 (52.2%)	51 (92.7%)	75 (74.3%)
	1		16 (34.8%)	4 (7.3%)	20 (19.8%)
	2		6 (13.0%)	0 (0.0%)	6 (5.9%)
Months of TNFi treatment in treated patients	82		84.0 (48.0, 132.0)	19.0 (1.0, 43.0)	51.0 (1.0, 132.0)
Uveitis	101		10 (21.7%)	12 (21.8%)	22 (21.8%)
Psoriasis	99		5 (11.1%)	2 (3.7%)	7 (7.1%)
IBD	99		3 (6.7%)	4 (7.4%)	7 (7.1%)

Continuous variables are described by their median and their minimum and maximum values (between brackets), while absolute and percentages are showed for categorical variables. *HLA-B27*, human leucocyte antigen B27; *AS*, ankylosing spondylitis; *BMI*, body mass index; *CRP*, C reactive protein; *BASDAI*, Bath Ankylosing Spondylitis Disease Activity Index; *ASDAS-CRP*, Ankylosing Spondylitis Disease Activity Score; *BASFI*, Bath Ankylosing Spondylitis Functional Index; *BASMI*, Bath Ankylosing Spondylitis Metrology Index; *mSASSS*, modified Stoke Ankylosing Spondylitis Spine Score; *NSAID*, non-steroidal anti-inflammatory drug; *TNFi*, tumor necrosis factor inhibitor; *IBD*, inflammatory bowel disease

Agreement between both readers was “excellent” for mSASSS status score (ICC 0.99 and 0.98, at inclusion and follow-up, respectively) and “moderate” for the change of the mSASSS score by ≥2 point (Cohen’s kappa 0.53). The Bland and Altman plots illustrating inter-reader reliability are presented in Supplementary Figure S1. More inter-reader reliability data is available in Supplementary Table S5. The mean (SD) time between radiographs was 3.5 (1.0) years, the change score between inclusion and follow-up was 1.98 (0.83), and the SDC of progression was 2.26 mSASSS units. Progression by ≥ 2 units in the total mSASSS score during follow-up was observed in 30 patients (29.7%).

**Fig. 2:**
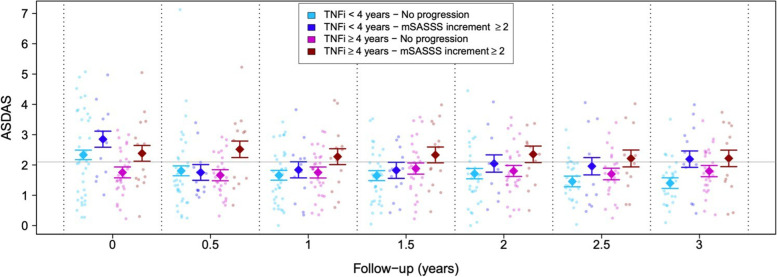
Association between disease activity measured by ASDAS-CRP every 6 months with radiographic progression. Radiographic progression was defined as an increase in the mSASSS score at ≥2 points in patients under TNFi treatment during a follow-up period of 3 years. Bold dots and intervals represent means and ± their corresponding standard errors, as provided by a mixed-effects linear model (no other covariates included)

Among patients receiving long-term treatment, significant mean ASDAS differences were found at baseline (i.e., at time of inclusion) between progressors and non-progressors (mean difference = 0.63; *p*-value = 0.046) (Fig. 2). The magnitude of this difference, while not statistically significant, was preserved after adjusting for clinical potential confounders (mean difference of 0.61, *p*-value = 0.058). Importantly, these patients maintained essentially the same baseline ASDAS levels during the 3-year follow-up with an adjusted mean difference ranging from −0.14 to 0.16 (Fig. 2). Based on this observation, we performed further analyses in which ASDAS values were averaged across all time points (Table 2). In this comparison, averaged ASDAS was significantly higher in progressors compared to non-progressors mean difference = 0.56, *p*-value = 0.027), even after controlling for potential confounders (mean difference = 0.57, *p*-value = 0.031, Table 2). Importantly, averaged ASDAS remained above the 2.1 threshold (2.33) that indicates high disease activity in patients who progressed. In contrast, this estimation was within the low activity range (1.76) for non-progressors (Supplementary Figure S2).

**Table 2:** Disease activity differences between patients under long-term TNFi treatment with and without radiographic progression

Univariable analysis			
	Progressors mean (SE)	Non-progressors mean (SE)	Difference
**ASDAS**	2.33 (0.21)	1.76 (0.14)	0.56 *p* = 0.027
**BASDAI**	3.86 (0.42)	2.97 (0.29)	0.89 *p* = 0.085
**CRP**	1.40 (0.09)	1.21 (0.06)	0.19 *p* = 0.087
Multivariable model			
	Progressors (SE)	Non-progressors (SE)	Difference
**ASDAS**	2.38 (0.23)	1.81 (0.14)	0.57 *p* = 0.031
**BASDAI**	3.83 (0.46)	3.03 (0.28)	0.80 *p* = 0.144
**CRP**	1.41 (0.11)	1.22 (0.07)	0.19 *p* = 0.130

Results derived from a mixed-effect linear model with no other covariates (univariable analysis) or including potential confounders factors collected at time of recruitment (multivariable analysis): length of the radiographic interval, gender, age, smoking habit, radiographic sacroiliitis, mSASSS average, presence of syndemosphytes, HLAB27, and time from symptomatology onset. Statistical significance was assessed using Wald tests derived from the model. *SE*, standard error; *p*, *p*-value; *CRP*, C-reactive protein; *BASDAI*, Bath Ankylosing Spondylitis Disease Activity Index; *ASDAS-CRP*, Ankylosing Spondylitis Disease Activity

In the no long-term TNFi treatment group (<4 years), similar ASDAS differences at inclusion were seen in progressors and non-progressors (mean difference = 0.52; *p*-value = 0.093) (Fig. 2). As expected, ASDAS scores in this group were higher than those observed for patients on long-term treatment, irrespective of their progression status (mean difference = −0.52; *p*-value = 0.018). Nonetheless, these patients experienced a substantial decrease in their ASDAS scores after TNFi treatment was prescribed at recruitment, observed as early as after 6 months of follow-up (mean decrease of −0.53, *p*-value <0.001 in non-progressors; mean decrease of −1.10, *p*-value <0.001 in progressors). At the end of follow-up, this decrease in disease activity induced by TNFi therapy was conserved, or even accentuated in patients without radiographic progression (additional mean decrease of 0.41 during follow-up compared to the first time point, *p*-value = 0.015). On the other hand, patients with radiographic progression did not maintain this substantial decrease in ASDAS during follow-up (mean increase of 0.44 compared to first time point, *p*-value = 0.083). These differences in ASDAS profiles throughout follow-up were evident after controlling for clinical variables potentially linked to disease progress (additional mean decrease of 0.30, p-value = 0.074 in the non-progressors group; mean increase of 0.41, *p*-value 0.100 in the progressors group, compared to the first time point).

Nevertheless, it was not until the third year of follow-up that significant ASDAS differences were found between progression status in patients not treated in the long-term group, with progressors showing a higher level of ASDAS compared to non-progressors at the end of follow-up (mean difference of 0.79, *p*-value = 0.014). At this time point, only 15% (4 out of 27) with no radiographic progression showed a high level of disease activity (ASDAS ≥ 2.1), while this percentage rose to 50% (6 out of 12) in patients with radiographic progression (OR = 18.39, *p*-value = 0.037). Despite the small sample size available for this comparison, the above differences persisted after controlling statistically for potential confounders, although they were not statistically significant at the selected threshold (mean difference: 0.57, *p*-value = 0.093; OR = 17.5, *p*-value = 0.057). Furthermore, an increase of 1 point in the ASDAS score at this time point (3 years) was associated with a 16% increase in the mSASSS, both compared to values obtained at recruitment (*p*-value = 0.021).

Although similar patterns were found when analyzing disease activity with BASDAI score and CRP serum levels, we did not observe significant mean differences between progressors and non-progressors in long-term TNFi-treated patients (Table 2). Additionally, in contrast to ASDAS findings, in the third year of follow-up, no significant differences were found between progressors and non-progressors in patients not under long-term TNFi treatment (Supplementary Figure S3).

## Discussion

In this study, we evaluated the association between disease activity and radiographic spinal progression in axSpA patients receiving TNFi who were included in a Spanish national registry (REGISPONSERBIO). Our results showed that disease activity measured by ASDAS is clearly associated with radiographic spinal progression in patients on TNFi treatment.

The duration of biologic treatment is an important factor in assessing its effect on spinal progression in axSpA. As previous studies showed a potential association between the duration of TNFi treatment and risk of progression [7, 9, 21], with no TNFi effect being observed within a follow-up period of 2 years [9, 22]. Moreover, the potential long-term effect of TNFi on new bone formation is thought to be due to an effective suppression of inflammation. In this regard, new evidence suggests that inflammatory changes are followed by the replacement of subchondral bone marrow by repairing tissue which then stimulates osteoblasts, leading to new bone formation [1]. Based on these observations, the association between disease activity and radiographic spinal progression was evaluated separately for patients receiving long-term treatment (TNFi ≥4 years) and patients treated for a shorter period. Our results showed that patients on long-term treatment with a mean sustained average of low disease activity measured by ASDAS over time show significantly less progression rates than those with high ASDAS averages. Conversely, in patients treated for less than 4 years who experimented radiographic progression presented a progressive increase of ASDAS mean score compared with those without progression. Such differences, however, did not achieve statistical significance until the third year of follow-up. Hence, our data supports previous studies that suggest a slowdown effect on radiographic progression associated to the maintenance of disease control in patients with axSpA treated with TNFi [3, 22].

To our knowledge, this is the first study suggesting that low disease activity is an appropriate target in the inhibition of radiographic spinal progression. As stated in the recent ASAS-EULAR management recommendations for axSpA, a treat-to-target approach is recommended [10, 23]. Nevertheless, there is no absolute consensus about which specific target to use. Molnar et al. [9] suggest that inactive disease (ASDAS <1.3) might be an adequate target for the inhibition of spinal radiographic progression. However, it might be quite challenging to achieve inactive disease in standard clinical practice, as only 25–35% of patients achieve inactive disease status, according to previously published studies [18, 24]. These observations are in line with those of our own cohort, in which only 23% of patients had inactive disease at the time of recruitment, while 52.1% of patients achieved low disease activity. Nevertheless, our findings suggest that low disease activity (ASDAS <2.1) might be an appropriate target for preventing radiographic progression and a more reasonable treatment objective to achieve in clinical practice.

In our data, disease activity measured by BASDAI and CRP showed similar trends to ASDAS, although the association with progression status was weaker and did not show statistical significance. These findings are in line with previous publications, where despite an acceptable concordance between patient groups defined by BASDAI ≥4 and ASDAS ≥2.1 [25, 26], ASDAS is the preferred tool for assessing disease activity [10]. As reported by previous authors, a longitudinal relationship between ASDAS and subsequent syndesmophyte formation was found, while such a relationship between BASDAI and syndesmophytes was far weaker [3, 27]. Furthermore, ASDAS shows a better correlation than BASDAI when measurements of disease activity assessed by patients and physicians are compared [28]. Finally, ASDAS cutoffs for disease activity and response criteria are well established and are based on a thorough validation process, in contrast to the BASDAI scale where thresholds have been defined in a more arbitrarily manner [24].

Our study had several limitations. First, not all REGISPONSERBIO patients could be included in the current study due to the unavailability of suitable radiographs. This point has negatively affected the final number of patients available for inclusion and only 30 patients with radiographic progression were available for analyses (15 progressors vs 40 non-progressors in the long-term treatment group; 15 progressors vs 31 non-progressors in the not long-term treated patients). Secondly, conventional radiography might be an insensitive tool for detecting radiographic spinal progression compared to other imaging techniques. Low-dose CT has the potential to become the primary method used to detect radiographic spinal progression in axSpA in the future, as it has been shown to detect more progression in the form of new and growing syndesmophytes in patients with r-axSpA [29]. Currently, conventional radiography requires a radiographic interval of at least 2 years to detect a relevant change, so long-term treatment is necessary before radiographic progression can be detected. Another possible issue is the extrapolation of our results to the study population, as only 101 patients out from 257 that are part of the REGISPONSERBIO cohort were included in the study based on the availability of full sets of radiographs (lumbar and cervical lateral view) and clinical data. Nevertheless, we found only minor differences regarding variables that could influence disease activity and radiographic progression, which discards a major impact on the results due to a selection bias. Finally, although readers were blinded to all clinical data, radiographic scoring was performed with readers being aware of the chronologic order. However, this approach appears to be more sensitive for detecting change than reading with paired time order [30].

One of the strengths of our study is the prospective nature of the cohort used in our study, which provided disease activity data every 6 months over a follow-up period of 3 years. This amount of disease activity data offered an accurate picture of disease status in patients treated with TNFi. Additionally, this design allowed the use of statistical tools to analyze the data as an experiment of repeated measures (linear-mixed effects model). Finally, disease activity was assessed with ASDAS-CRP, BASDAI score, and CRP serum levels, allowing comparison in terms of radiographic implications.

## Conclusions

Patients receiving TNFi with a mean sustained low disease activity over time measured by ASDAS presented less radiographic progression rates than those with high disease activity. The benefit of maintaining ASDAS at low levels becomes apparent after long-term treatment with TNFi (≥4 years). Moreover, our data emphasize the use of ASDAS as the preferred tool to assess disease activity, and suggest that low disease activity (<2.1) is an acceptable threshold as a therapeutic objective in a treat-to-target strategy to prevent radiographic progression.

## Supplementary Information

**Additional file 1: Supplementary Table S1**. Baseline characteristics of the 101 patients included in the study by baseline TNFi treatment status and radiographic progression. **Supplementary Table S2.** Baseline characteristics of the 101 patients included in the study by radiographic progression. **Supplementary Table S3**. Baseline characteristics of the 156 REGISPONSERBIO patients not included in the study. **Supplementary Table S4.** Comparison of baseline characteristics at first visit between patients included (101) and not included (156) in the study. **Supplementary Table S5.** Inter-observer reliability. **Supplementary Figure S1** Blat and Altman Pot, inter-reader reliability**. Supplementary Figure S2.** Boxplot of the ASDAS values averaged across all time points in patients under long-term TNFi treatment. **Supplementary Figure S3.** Association between disease activity measured by BASDAI (a) and CRP(b) every 6 months with radiographic progression defined as an increase in the mSASSS score at ≥2 points in patients unter TNFi treatment during a follow-up period of three years. Bold dots and intervals represent means and +/- their corresponding standard errors provided a the mixed-effects linear model (no other covariates included).

## Data Availability

The data that support the findings of this study are available from the corresponding author upon reasonable request.

## Abbreviations

AxSpA: Axial spondyloarthritis
Nr-axSpA: Non-radiographic SpA
R-axSpA: Radiographic SpA
AS: Ankylosing Spondylitis
TNFi: TNF inhibitors
mSASS: Modified Stoke Ankylosing Spondylitis Spine Score
ASDAS: Ankylosing Spondylitis Disease Activity Score
BASDAI: Bath Ankylosing Spondylitis Disease Activity Index
BASFI: Bath Ankylosing Spondylitis Functional Index
VC: Vertebral corner
SDC: Smallest detectable change
CRP: C-reactive protein
SD: Standard deviation
OR: Odds ratio
ICC: Intraclass correlation coefficient
