# Supplementary material for: Sustained low disease activity measured by ASDAS slow radiographic spinal progression in axial spondyloarthritis patients treated with TNF-inhibitors: data from REGISPONSERBIO
Source: Arthritis Res Ther. 2022 Jan 21;24:30. doi: 10.1186/s13075-021-02695-5 (PMC8780330; doi:10.1186/s13075-021-02695-5)
Supplement: Supplementary file 1 — Additional file 1: Supplementary Table S1. Baseline characteristics of the 101 patients included in the study by baseline TNFi treatment status and radiographic progression. Supplementary Table S2. Baseline characteristics of the 101 patients included in the study by radiographic progression. Supplementary Table S3. Baseline characteristics of the 156 REGISPONSERBIO patients not included in the study. Supplementary Table S4. Comparison of baseline characteristics at first visit between patients included (101) and not included (156) in the study. Supplementary Table S5. Inter-observer reliability. Supplementary Figure S1 Blat and Altman Pot, inter-reader reliability. Supplementary Figure S2. Boxplot of the ASDAS values averaged across all time points in patients under long-term TNFi treatment. Supplementary Figure S3. Association between disease activity measured by BASDAI (a) and CRP(b) every 6 months with radiographic progression defined as an increase in the mSASSS score at ≥2 points in patients unter TNFi treatment during a follow-up period of three years. Bold dots and intervals represent means and +/- their corresponding standard errors provided a the mixed-effects linear model (no other covariates included). [file 13075_2021_2695_MOESM1_ESM.docx]

**Supplementary Material**

**Supplementary Table S1.** Baseline characteristics of the 101 patients included in the study by baseline TNFi treatment status and radiographic progression.

|  | **N** | | TNFi >= 4 years No progression N=31 (30.7%) | TNFi >= 4 years MSASSS increment >= 2 N=15 (14.9%) | **TNFi < 4 years No progression N=40 (39.6%)** | **TNFi < 4 years MSASSS increment >= 2  N=15 (14.9%)** | **All N=101** |
| --- | --- | --- | --- | --- | --- | --- | --- |
| Age, years | 101 | | 44 | 55 | 43 | 54 | 46 |
|  | 101 | | (25.00, 68.00) | (36.00, 75.00) | (21.00, 69.00) | (35.00, 75.00) | (21.00, 75.00) |
| Female, sex % | 101 | | 7 | 1 | 8 | 3 | 19 |
|  | 101 | | (22.6%) | (6.7%) | (20.0%) | (20.0%) | (18.8%) |
| HLA-B27 positive, % | 99 | | 29 | 14 | 32 | 11 | 86 |
|  | 99 | | (96.7%) | (93.3%) | (80.0%) | (78.6%) | (86.9%) |
| AS, % | 101 | | 26 | 15 | 30 | 15 | 86 |
|  | 101 | | (83.9%) | (100.0%) | (75.0%) | (100.0%) | (85.1%) |
| BMI | 95 | | 26.67 | 26.59 | 24.4 | 27.22 | 26.02 |
|  | 95 | | (19.78, 40.83) | (22.79, 36.21) | (19.37, 32.03) | (21.89, 31.88) | (19.37, 40.83) |
| Current smokers, % | 101 | | 9 | 7 | 12 | 3 | 31 |
|  | 101 | | (29.0%) | (46.7%) | (30.0%) | (20.0%) | (30.7%) |
| Symptom duration, years | 97 | | 15 | 30 | 10 | 14 | 15 |
|  | 97 | | (2.00, 39.00) | (14.00, 52.00) | (0.00, 50.00) | (1.00, 46.00) | (0.00, 52.00) |
| CRP (mg/L) | 94 | | 2.3 | 6.6 | 2.4 | 5.75 | 3.35 |
|  | 94 | | (0.10, 26.00) | (1.00, 22.00) | (0.00, 38.20) | (1.00, 88.70) | (0.00, 88.70) |
| CRP < 5 | 94 | | 23 | 4 | 21 | 5 | 53 |
|  | 94 | | (74.2%) | (33.3%) | (56.8%) | (35.7%) | (56.4%) |
| BASDAI (0-10) | 97 | | 2.9 | 4 | 4.5 | 3.2 | 3.2 |
|  | 97 | | (0.20, 5.50) | (0.20, 8.60) | (0.40, 8.70) | (0.70, 8.80) | (0.20, 8.80) |
| BASDAI < 4 | 97 | | 24 | 6 | 16 | 8 | 54 |
|  | 97 | | (77.4%) | (42.9%) | (43.2%) | (53.3%) | (55.7%) |
| ASDAS-CRP | 96 | | 1.63 | 2.26 | 2.16 | 2.82 | 2.06 |
|  | 96 | | (0.23, 3.15) | (0.30, 5.05) | (0.27, 5.07) | (1.01, 4.97) | (0.23, 5.07) |
| Low disease (ASDAS < 2.1), % | 96 | | 21 | 7 | 18 | 4 | 50 |
|  | 96 | | (70.0%) | (50.0%) | (46.2%) | (30.8%) | (52.1%) |
| Inactive disease (ASDAS <1.3), % | 96 | | 9 | 1 | 12 | 1 | 23 |
|  | 96 | | (30.0%) | (7.1%) | (30.8%) | (7.7%) | (24.0%) |
| BASFI (0-10) | 98 | | 2.5 | 5.8 | 4.15 | 5.2 | 3.85 |
|  | 98 | | (0.00, 7.20) | (0.00, 9.10) | (0.00, 9.30) | (1.30, 9.30) | (0.00, 9.30) |
| BASMI (0-10) | 82 | | 2.2 | 4.44 | 2.23 | 4.64 | 2.87 |
|  | 82 | | (0.54, 5.85) | (0.99, 7.27) | (0.57, 4.24) | (1.08, 6.41) | (0.54, 7.27) |
| MSASSS (0-72) | 101 | | 3 | 21.5 | 0.5 | 22.5 | 5 |
|  | 101 | | (0.00, 72.00) | (5.00, 63.00) | (0.00, 66.00) | (1.50, 63.00) | (0.00, 72.00) |
| Syndesmophytes present, % | 101 | | 12 | 14 | 12 | 13 | 51 |
|  | 101 | | (38.7%) | (93.3%) | (30.0%) | (86.7%) | (50.5%) |
| On NSAID treatment, % | 99 | | 18 | 5 | 27 | 7 | 57 |
|  | 99 | | (58.1%) | (35.7%) | (67.5%) | (50.0%) | (57.6%) |
| On TNFi treatment, % | 100 | | 31 | 14 | 28 | 9 | 82 |
|  | 100 | | (100.0%) | (100.0%) | (70.0%) | (60.0%) | (82.0%) |
| Number of previous TNFi | 101 | 0 | 18 | 6 | 36 | 15 | 75 |
|  | 101 |  | (58.1%) | (40.0%) | (90.0%) | (100.0%) | (74.3%) |
|  |  | 1 | 11 | 5 | 4 | 0 | 20 |
|  | 101 |  | (35.5%) | (33.3%) | (10.0%) | (0.0%) | (19.8%) |
|  |  | 2 | 2 | 4 | 0 | 0 | 6 |
|  | 101 |  | (6.5%) | (26.7%) | (0.0%) | (0.0%) | (5.9%) |
| Months of TNFi treatment in treated patients | 82 | | 79 | 93.5 | 20 | 18 | 51 |
|  | 82 | | (48.00, 132.00) | (48.00, 124.00) | (1.00, 43.00) | (3.00, 43.00) | (1.00, 132.00) |
| Uveitis | 101 | | 6 | 4 | 9 | 3 | 22 |
|  | 101 | | (19.4%) | (26.7%) | (22.5%) | (20.0%) | (21.8%) |
| Psoriasis | 99 | | 3 | 2 | 1 | 1 | 7 |
|  | 99 | | (9.7%) | (14.3%) | (2.6%) | (6.7%) | (7.1%) |
| IBD | 99 | | 3 | 0 | 4 | 0 | 7 |
|  | 99 | | (9.7%) | (0.0%) | (10.3%) | (0.0%) | (7.1%) |

Continuous variables are described by their median and their minimum and maximum values (between brackets), while absolute and percentages are showed for categorical variables. **HLA-B27:** human leucocyte antigen B27; **AS;** Ankylosing Spondlitis; **BMI:** body mass index; **CRP:** C reactive protein; **BASDAI:** Bath Ankylosing Spondylitis Disease Activity Index; **ASDAS-CRP:** Ankylosing Spondylitis Disease Activity Score; **BASFI:** Bath Ankylosing Spondylitis Functional Index; **BASMI:** Bath Ankylosing Spondylitis Metrology Index; **mSASSS:** modified Stoke Ankylosing Spondylitis Spine Score; **NSAID:** non-steroidal anti-inflammatory drug; **TNFi:** tumour necrosis factor inhibitor; **IBD**: Inflammatory bowel disease.

**Supplementary Table S2.** Baseline characteristics  of the 101 patients included in the study by radiographic progression.

|  | **N** | | **No progression**  **n=71 (70.3%)** | **mSASSS increment >= 2**  **n=30 (29.7%)** | **All**  **n=101** |
| --- | --- | --- | --- | --- | --- |
| Age, years | 101 | | 43 | 54 | 46 |
|  | 101 | | (21.00, 69.00) | (35.00, 75.00) | (21.00, 75.00) |
| Female, sex % | 101 | | 15 | 4 | 19 |
|  | 101 | | (21.1%) | (13.3%) | (18.8%) |
| HLA-B27 positive, % | 99 | | 61 | 25 | 86 |
|  | 99 | | (87.1%) | (86.2%) | (86.9%) |
| AS, % | 101 | | 56 | 30 | 86 |
|  | 101 | | (78.9%) | (100.0%) | (85.1%) |
| BMI | 95 | | 25.35 | 27.22 | 26.02 |
|  | 95 | | (19.37, 40.83) | (21.89, 36.21) | (19.37, 40.83) |
| Current smokers, % | 101 | | 21 | 10 | 31 |
|  | 101 | | (29.6%) | (33.3%) | (30.7%) |
| Symptom duration, years | 97 | | 13 | 19 | 15 |
|  | 97 | | (0.00, 50.00) | (1.00, 52.00) | (0.00, 52.00) |
| CRP (mg/L) | 94 | | 2.3 | 6.4 | 3.35 |
|  | 94 | | (0.00, 38.20) | (1.00, 88.70) | (0.00, 88.70) |
| CRP < 5 | 94 | | 44 | 9 | 53 |
|  | 94 | | (64.7%) | (34.6%) | (56.4%) |
| BASDAI (0-10) | 97 | | 3.2 | 4 | 3.2 |
|  | 97 | | (0.20, 8.70) | (0.20, 8.80) | (0.20, 8.80) |
| BASDAI < 4 | 97 | | 40 | 14 | 54 |
|  | 97 | | (58.8%) | (48.3%) | (55.7%) |
| ASDAS-CRP | 96 | | 1.97 | 2.7 | 2.06 |
|  | 96 | | (0.23, 5.07) | (0.30, 5.05) | (0.23, 5.07) |
| Low disease (ASDAS < 2.1), % | 96 | | 39 | 11 | 50 |
|  | 96 | | (56.5%) | (40.7%) | (52.1%) |
| Inactive disease (ASDAS <1.3), % | 96 | | 21 | 2 | 23 |
|  | 96 | | (30.4%) | (7.4%) | (24.0%) |
| BASFI (0-10) | 98 | | 3.55 | 5.25 | 3.85 |
|  | 98 | | (0.00, 9.30) | (0.00, 9.30) | (0.00, 9.30) |
| BASMI (0-10) | 82 | | 2.21 | 4.64 | 2.87 |
|  | 82 | | (0.54, 5.85) | (0.99, 7.27) | (0.54, 7.27) |
| MSASSS (0-72) | 101 | | 1.5 | 22 | 5 |
|  | 101 | | (0.00, 72.00) | (1.50, 63.00) | (0.00, 72.00) |
| Syndesmophytes present, % | 101 | | 24 | 27 | 51 |
|  | 101 | | (33.8%) | (90.0%) | (50.5%) |
| On NSAID treatment, % | 99 | | 45 | 12 | 57 |
|  | 99 | | (63.4%) | (42.9%) | (57.6%) |
| On TNFi treatment, % | 100 | | 59 | 23 | 82 |
|  | 100 | | (83.1%) | (79.3%) | (82.0%) |
| Number of previous TNFi | 101 | 0 | 54 | 21 | 75 |
|  | 101 |  | (76.1%) | (70.0%) | (74.3%) |
|  |  | 1 | 15 | 5 | 20 |
|  |  |  | (21.1%) | (16.7%) | (19.8%) |
|  |  | 2 | 2 | 4 | 6 |
|  |  |  | (2.8%) | (13.3%) | (5.9%) |
| Months of TNFi treatment in treated patients | 82 | | 49.00  (1.00, 132.00) | 62.00  (3.00, 124.00) | 51.00  (1.00, 132.00) |
| Uveitis | 101 | | 15  (21.1%) | 7  (23.3%) | 22  (21.8%) |
| Psoriasis | 99 | | 4 | 3 | 7 |
|  | 99 | | (5.7%) | (10.3%) | (7.1%) |
| IBD | 99 | | 7 | 0 | 7 |
|  | 99 | | (10.0%) | (0.0%) | (7.1%) |

Continuous variables are described by their median and their minimum and maximum values (between brackets), while absolute and percentages are showed for categorical variables. **HLA-B27:** human leucocyte antigen B27; **AS;** Ankylosing Spondlitis; **BMI:** body mass index; **CRP:** C reactive protein; **BASDAI:** Bath Ankylosing Spondylitis Disease Activity Index; **ASDAS-CRP:** Ankylosing Spondylitis Disease Activity Score; **BASFI:** Bath Ankylosing Spondylitis Functional Index; **BASMI:** Bath Ankylosing Spondylitis Metrology Index; **mSASSS:** modified Stoke Ankylosing Spondylitis Spine Score; **NSAID:** non-steroidal anti-inflammatory drug; **TNFi:** tumour necrosis factor inhibitor; **IBD**: Inflammatory bowel disease.

**Supplementary Table S3**. Baseline characteristics of the 156 REGISPONSERBIO patients not included in the study.

|  | N | | TNFi treatment > 4years  n=61  (39.9%) | **TNFi treatment < 4years**  **n=92**  **(60.1%)** | **All**  **n=153** |
| --- | --- | --- | --- | --- | --- |
| Age, years | 144 | | 52 | 48.5 | 49 |
|  | 144 | | (26.00, 73.00) | (19.00, 80.00) | (19.00, 80.00) |
| Female, sex % | 156 | | 12 | 26 | 38 |
|  | 156 | | (19.7%) | (28.3%) | (24.4%) |
| HLA-B27 positive, % | 153 | | 50 | 66 | 119 |
|  | 153 | | (82.0%) | (74.2%) | (77.8%) |
| AS, % | 156 | | 50 | 64 | 116 |
|  | 156 | | (82.0%) | (69.6%) | (74.4%) |
| BMI | 139 | | 26.26 | 26.26 | 26.26 |
|  | 139 | | (18.69, 37.96) | (17.85, 36.42) | (17.85, 37.96) |
| Current smokers, % | 155 | | 15 | 27 | 43 |
|  | 155 | | (25.0%) | (29.3%) | (27.7%) |
| Symptom duration, years | 142 | | 22.09 | 12.64 | 17.75 |
|  | 142 | | (5.37, 49.10) | (0.93, 51.51) | (0.93, 51.51) |
| CRP (mg/L) | 153 | | 3 | 5 | 3.59 |
|  | 153 | | (0.00, 77.00) | (0.00, 40.10) | (0.00, 77.00) |
| CRP < 5 | 153 | | 42 | 47 | 92 |
|  | 153 | | (71.2%) | (51.6%) | (60.1%) |
| BASDAI (0-10) | 133 | | 3.3 | 5.6 | 4.6 |
|  | 133 | | (0.00, 90.00) | (0.30, 30.00) | (0.00, 90.00) |
| BASDAI < 4 | 133 | | 33 | 24 | 59 |
|  | 133 | | (63.5%) | (30.4%) | (44.4%) |
| ASDAS-CRP | 136 | | 2.15 | 2.74 | 2.42 |
|  | 136 | | (0.00, 4.26) | (0.26, 5.11) | (0.00, 5.11) |
| Low disease (ASDAS < 2.1), % | 136 | | 24 | 29 | 55 |
|  | 136 | | (48.0%) | (34.5%) | (40.4%) |
| Inactive disease (ASDAS <1.3), % | 136 | | 13 | 10 | 24 |
|  | 136 | | (26.0%) | (11.9%) | (17.6%) |
| BASFI (0-10) | 154 | | 2.5 | 4.7 | 3.7 |
|  | 154 | | (0.00, 9.80) | (0.00, 10.00) | (0.00, 10.00) |
| BASMI (0-10) | 77 | | 3.8 | 3 | 3.4 |
|  | 77 | | (1.00, 6.80) | (1.00, 7.80) | (1.00, 7.80) |
| On NSAID treatment, % | 155 | | 31 | 65 | 98 |
|  | 155 | | (51.7%) | (70.7%) | (63.2%) |
| On TNFi treatment, % | 156 | | 61 | 36 | 100 |
|  | 156 | | (100.0%) | (39.1%) | (64.1%) |
| Number of previous TNFi | 153 | 0 | 82  (89.1%) | 33  (54.1%) | 118  (75.6%) |
|  |  | 1 | 7  (7.6%) | 20  (32.8%) | 27  (17.3%) |
|  |  | 2 | 1  (1.1%) | 6  (9.8%) | 8  (5.1%) |
|  |  | 3 | 6 | 2  (3.3%) | 3  (1.9%) |
| Biological treatment at recruitment | 151 | Adalimumab | 17 | 27 | 46 |
|  | 151 |  | (28.8%) | (30.3%) | (30.5%) |
|  |  | Etanercept | 23 | 29 | 52 |
|  |  |  | (39.0%) | (32.6%) | (34.4%) |
|  |  | Golimumab | 3 | 25 | 29 |
|  |  |  | (5.1%) | (28.1%) | (19.2%) |
|  |  | Infliximab | 16 | 8 | 24 |
|  |  |  | (27.1%) | (9.0%) | (15.9%) |
| Months of TNFi treatment in treated patients | 153 | | 81.02 | 0 | 32.69 |
|  | 153 | | (48.76, 156.71) | (0.00, 46.98) | (0.00, 156.71) |
| Uveitis | 155 | | 21 | 22 | 45 |
|  | 155 | | (35.0%) | (23.9%) | (29.0%) |
| Psoriasis | 155 | | 8 | 5 | 13 |
|  | 155 | | (13.3%) | (5.4%) | (8.4%) |
| IBD | 154 | | 5 | 12 | 17 |
|  | 154 | | (8.3%) | (13.2%) | (11.0%) |

Continuous variables are described by their median and their minimum and maximum values (between brackets), while absolute and percentages are showed for categorical variables. **HLA-B27:** human leucocyte antigen B27; **AS;** Ankylosing Spondlitis; **BMI:** body mass index; **CRP:** C reactive protein; **BASDAI:** Bath Ankylosing Spondylitis Disease Activity Index; **ASDAS-CRP:** Ankylosing Spondylitis Disease Activity Score; **BASFI:** Bath Ankylosing Spondylitis Functional Index; **BASMI:** Bath Ankylosing Spondylitis Metrology Index; **mSASSS:** modified Stoke Ankylosing Spondylitis Spine Score; **NSAID:** non-steroidal anti-inflammatory drug; **TNFi:** tumour necrosis factor inhibitor; **IBD**: Inflammatory bowel disease.

**Supplementary Table S4.** Comparison of baseline characteristics at first visit between patients included (101) and not included (156) in the study.

|  | | Included | Excluded | P-value |
| --- | --- | --- | --- | --- |
|  |  | N=101 | N=156 |  |
| Age, years | | 46.00 (21.00, 75.00) | 49.00 (19.00, 80.00) | 0.0685 |
| Female, sex % | | 19 (18.8%) | 38 (24.4%) | 0.3569 |
| TNFi treatment > 4years | | 46 (45.5%) | 61 (39.9%) | 0.4361 |
| HLA-B27 positive, % | | 86 (86.9%) | 119 (77.8%) | 0.0970 |
| AS, % | | 86 (85.1%) | 116 (74.4%) | 0.0436 |
| BMI | | 26.02 (19.37, 40.83) | 26.26 (17.85 37.96) | 0.4698 |
| Current smokers, % | | 31 (30.7%) | 43 (27.7%) | 0.6727 |
| Symptom duration, years | | 15.00 (0.00, 52.00) | 17.75 (0.93, 51.51) | 0.2873 |
| CRP (mg/L) | | 3.35 (0.00, 88.70) | 3.59 (0.00, 77.00) | 0.8023 |
| CRP < 5 | | 53 (56.4%) | 92 (60.1%) | 0.5958 |
| BASDAI (0-10) | | 3.20 (0.20, 8.80) | 4.60 (0.00, 90.00) | 0.0433 |
| BASDAI < 4 | | 54 (55.7%) | 59 (44.4%) | 0.1091 |
| ASDAS-CRP | | 2.06 (0.23, 5.07) | 2.42 (0.00, 5.11) | 0.0831 |
| Low disease (ASDAS < 2.1), % | | 50 (52.1%) | 55 (40.4%) | 0.0837 |
| Inactive disease (ASDAS <1.3), % | | 23 (24.0%) | 24 (17.6%) | 0.2501 |
| BASFI (0-10) | | 3.85 (0.00, 9.30) | 3.70 (0.00, 10.00) | 0.7216 |
| BASMI (0-1 | | 2.87 (0.54, 7.27) | 3.40 (1.00, 7.80) | 0.1862 |
| On NSAID treatment, % | | 57 (57.6%) | 98 (63.2%) | 0.4289 |
| On TNFi treatment, % | | 82 (82.0%) | 100 (64.1%) | 0.0019 |
| Number of previous TNFi | 0 | 75 (74.3%) | 118 (75.6%) | 0.6357 |
|  | 1 | 20 (19.8%) | 27 (17.3%) |  |
|  | 3 | 0 (0.0%) | 8 (5.1%) |  |
| Months of TNFi treatment in treated patients | | 51.00 (1.00, 132.00) | 32.69 (0.00, 156.71) | 0.0005 |
| Uveitis | | 22 (21.8%) | 45 (29.0%) | 0.2446 |
| Psoriasis | | 7 (7.1%) | 13 (8.4%) | 0.8136 |
| IBD | | 7 (7.1%) | 17 (11.0%) | 0.3808 |

ontinuous variables are described by their median and their minimum and maximum values (between brackets), while absolute and percentages are showed for categorical variables. Statistical significance of differences between included and excluded patients are derived from Mann-Whitney and from Fisher tests for continuous and categorical variables, respectively. **HLA-B27**: human leucocyte antigen B27; **AS:** Ankylosing Spondlitis; **BMI:** body mass index; **CRP:** C reactive protein; **BASDAI:** Bath Ankylosing Spondylitis Disease Activity Index; **ASDAS-CRP**: Ankylosing Spondylitis Disease Activity Score; **BASFI:** Bath Ankylosing Spondylitis Functional Index; **BASMI:** Bath Ankylosing Spondylitis Metrology Index; **NSAID:** non-steroidal anti-inflammatory drug; **TNF**i: tumour necrosis factor inhibitor; **IBD**: Inflammatory bowel disease.

**Supplementary Table S5.** Inter-observer reliability

| ICC for the mSASSS status score baseline | 0.99 (0.979-0.990) |
| --- | --- |
| ICC for the mSASSS status score follow-up | 0.98 (0.972-0.987) |
| ICC for the change score | 0.65 (0.481-0.764) |
| Kappa for the development of new/growth of existing syndesmophytes | 0.61 (SD. 0.080) |
| Kappa for the development of new syndesmophytes | 0.60 (SD. 0.087) |
| Kappa for the change score ≥ 2 points | 0.53 (SD. 0.090) |

**MSASSS**: modified Stoke Ankylosing Spondylitis Spine Score; ICC= Intraclass correlation coefficient; SD= Standard Deviation

**
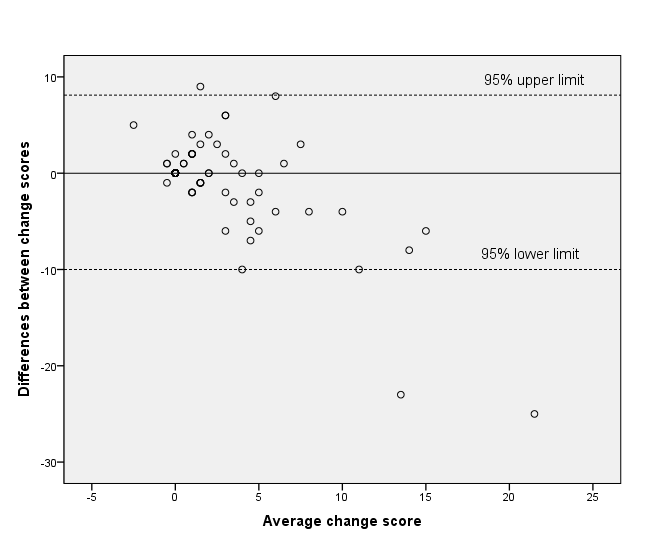
**

**S**

**Supplementary Figure S1** Blat and Altman Pot, inter-reader reliability


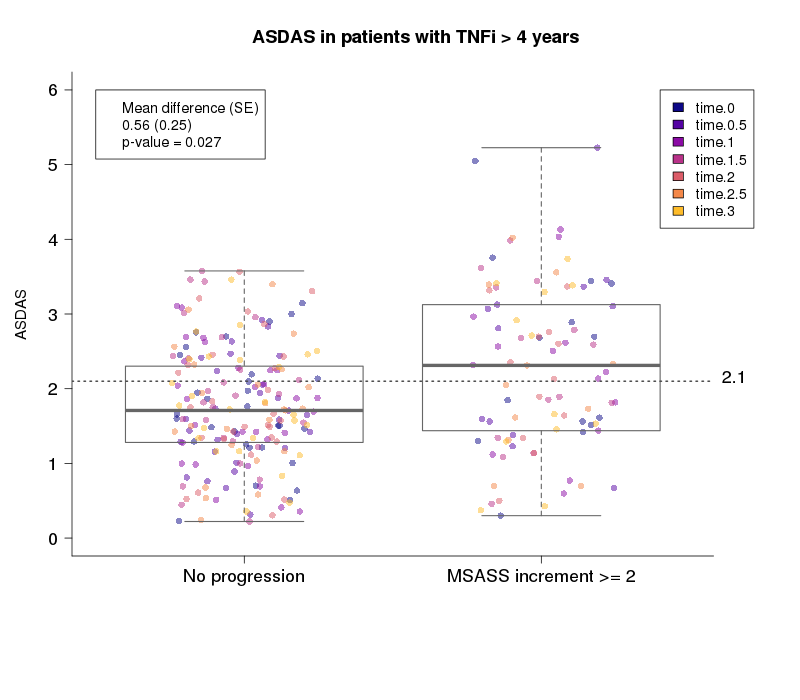


**Supplementary Figure S2.** Boxplot of the ASDAS values averaged across all time points in patients under long-term TNFi treatment.


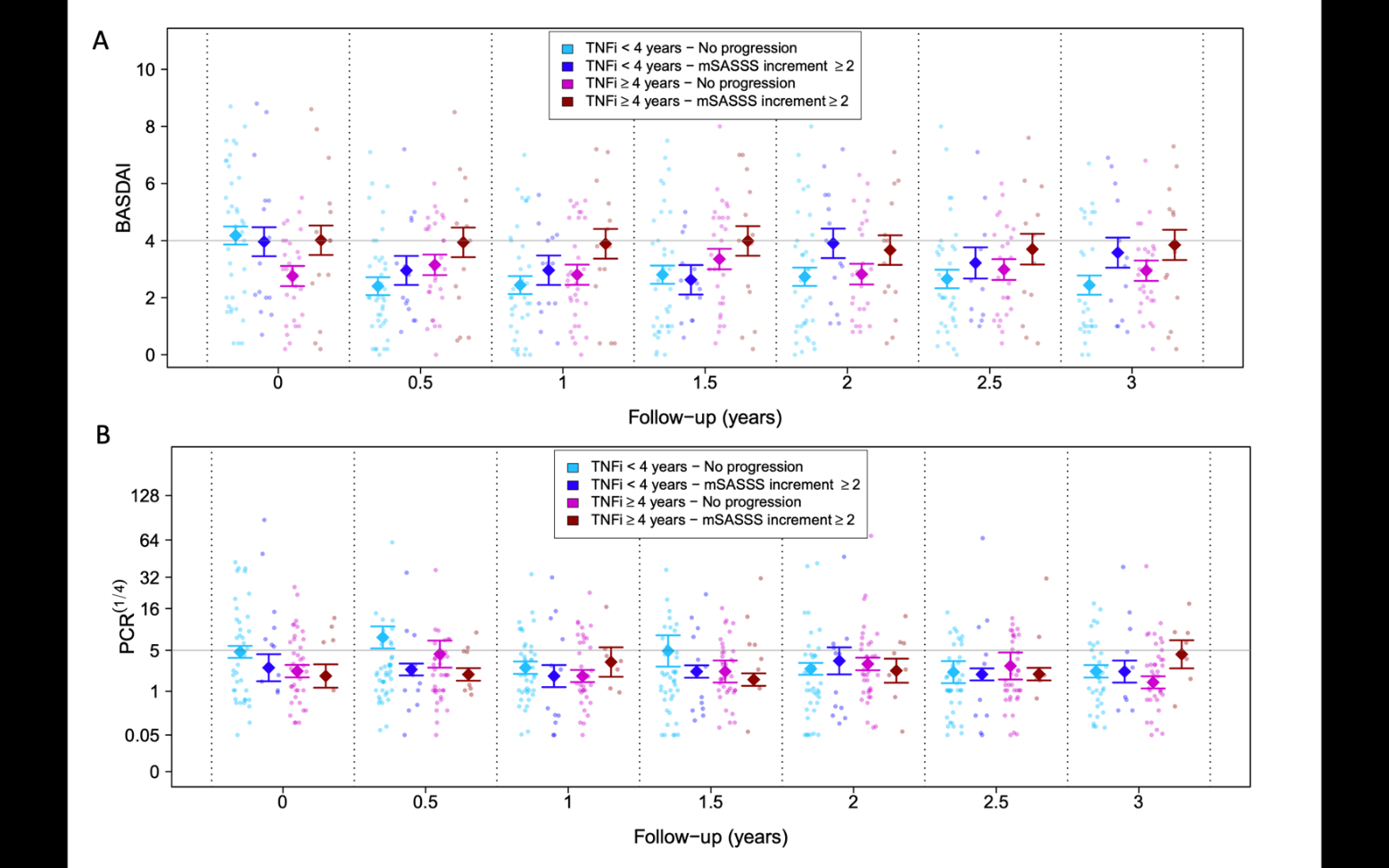


**Supplementary Figure S3.** Association between disease activity measured by BASDAI (a) and CRP(b) every 6 months with radiographic progression defined as an increase in the mSASSS score at ≥2 points in patients unter TNFi treatment during a follow-up period of three years. Bold dots and intervals represent means and +/- their corresponding standard errors provided a the mixed-effects linear model (no other covariates included).
